# Aristol

**Published:** 1891-08

**Authors:** 


### Selection.

### ARISTOL.

Dr. Alois Pollak (*Therapeut. Monatsh., XII., 1890,*) recommends aristol as an antiseptic, and as a remedy in various skin diseases, on the ground of experiments made in numerous cases. Inasmuch as this substance is insoluble in water, he employed it chiefly as a dusting powder, or in ethereal solutions or ointments. For obvious reasons, it cannot be utilized for disinfections of the hands, instruments, and the site of operation, or as an antiseptic during the operative procedure. On the other hand, it is very serviceable for the treatment of wounds after operations, or of neglected injuries. It has the great advantage of being effective in small quantities, so that wounds need only be covered with a thin layer; if desired, it may be diluted with sugar of milk. In all the cases treated with aristol by the author, the healing process took place without reaction. Fever never occurred, and if present before operation, it disappeared regularly within the first few days after its performance. There was an entire absence of pains, granulations were developed with remarkable rapidity, and formation of epithelium took place promptly. The period of healing was remarkably short.

The cases treated with aristol comprise the following:

1. Anal fistula; incision and curetting; tamponning with aristol gauze after previous application of the powder; cure in twenty days. 2. Abscess in the gluteal regions, of the size of a fist; incision and insufflation of aristol; healing accomplished in seven days. 3. Lymphadenitis of the neck (tuberculous ?); incision, extirpation, curetting; application of aristol in powder and tampons. Eight days later, fresh tubercle nodules in the granulations and cicatrix; after a second enucleation and the use of aristol, healing took place. 4. Periostitis of the mastoid process, incision, curetting followed by application of aristol powder; cure on the eleventh day. 5. Injury of the thigh by a rusty nail; wound has a discolored suspicious appearance, is covered by gray necrotic granulations, curetting and application of five per cent. aristol ointment; cured in fourteen days. 6. Necrotic ulcer in consequence of a contusion; incision of the undermined margins; application of aristol powder; healing ten days later.

In addition to these cases which are reported in full, the author mentions four minor operations for tumors, three extractions of foreign bodies, a wound inflicted by a pen dipped in ink, a lacerated wound of the scalp with exposure of the bone, three cases of purulent adenitis, three phlegmons, and several cases of buboes, all of which were successfully treated with aristol.

As regards the employment of aristol in the treatment of cutaneous diseases, he cites the following cases:

*Eczema scrofulosum.*— Among the ten cases, no curative effect was produced in six; in the remaining four it was slight. Hence, aristol is not to be recommended in this disease.

*Eczema marginatum.*— In this affection an excellent result was obtained from the use of a ten per cent. ointment with vaseline.

*Varicose ulcer.*— A five per cent ointment of aristol and vaseline was employed with advantage.

*Pernio.*— In the majority of cases, a ten per cent. solution of aristol in collodium and, later, aristol plaster, proved very effective.

Owing to its convenient manner of application, aristol seems especially applicable for the physician's use in his office practice.— *Deutsche Med. Zeitung*, April 2, 1891.

